# Combination immunotherapy with oncolytic vaccinia virus and checkpoint inhibitor following local tumor irradiation

**DOI:** 10.1186/2051-1426-2-S3-P112

**Published:** 2014-11-06

**Authors:** Boris Minev, Holbrook Kohrt, Mehmet Kilinc, Nanhai Chen, Audrey Feng, Maysam Pessian, Ulrike Geissinger, Erik Haefner, Desislava Tsoneva, Kliment Bozhilov, Idit Sagiv-Barfi, Xing Zhao, Narendiran Rajesekaran, Ronald Levy, Aladar Szalay

**Affiliations:** 1 University of California San Diego, San Diego, CA, USA; 2 Genelux Corporation, San Diego, CA, USA; 3 Stanford University, Stanford, CA, USA; 4 University of Wurzburg, Germany; 5 University of Hawaii, HI, USA

## 

**Figure 1:**
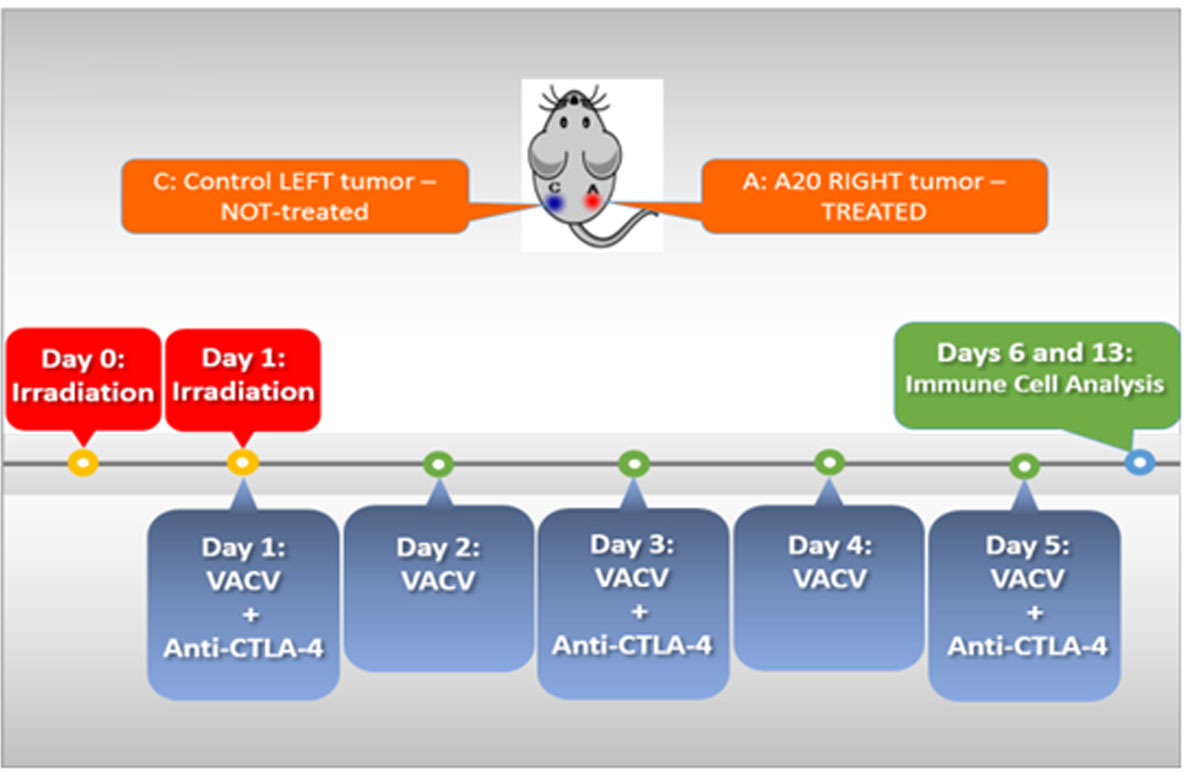


Oncolytic virotherapy is safe and clinically active in solid tumors, however its efficacy in hematologic malignancies as well as in combination with checkpoint inhibitors and radiation is unexplored. To simulate advanced lymphoma, A20 cells were injected subcutaneously on bilateral flanks of BALB/c mice and treatment initiated on day 17 to only the right flank tumor with local irradiation (Irr), intratumoral (i.t.) vaccinia virus (VACV) and i.t. anti-CTLA-4 mAb (Irr-VACV-CTLA4, Figure 1). The Irr-VACV-CTLA4 regimen was the most effective in eradicating or shrinking both treated and untreated tumors and extending survival, followed by the Irr-VACV regimen. Treatment with Irr-VACV-CTLA4, led to initially a mature, activated NK cell (KLRG1^+^CD27^+^) infiltrate (day 6 post-treatment) followed by a CD8^+^T cells infiltrate (day+13) in **treated tumors**. In contrast, treatment with VACV-CTLA4 led to activated NK cell accumulation (day +6) followed by a CD8^+ ^T cell infiltrate (day+13) in **non-treated tumors. **Importantly, CD8^+^CD44^hi ^T cells isolated from the blood and spleens of the treated mice showed functional specificity to A20 lymphoma cells, but not to MHC-matched tumor cells (CT26) in intra-cellular stains for IFN-γ. **Splenocyte-derived **A20-specific CD8^+^CD44^hi ^T cells were induced most efficiently in the Irr-VACV-CTLA4 regimen-treated mice, while **blood-derived **A20-specific CD8^+^CD44^hi ^T cells were induced most efficiently in the Irr-VACV regimen-treated mice. Viral plaque assays (VPA) showed lack of live viral particles in both treated and untreated tumors upon sacrificing mice 4 to 10 weeks after treatment initiation. Surprisingly, VPA assays identified live virus in the livers of Irr-VACV-CTLA4 regimen-treated mice, which paralleled a reduced metastatic load.

Our findings are the first to demonstrate the potential of combination immunotherapy with oncolytic viruses and checkpoint inhibitors in hematologic malignancies. The antitumor activity is attributed to the induction of an effective and specific immune response. This finding is corroborated by the significant infiltration with mature activated NK cells, followed by CD8^+ ^T cells, in both treated and untreated tumors. Importantly, the tumor-specific CD8^+ ^T cells showing a memory phenotype (CD44^hi^) suggest the effective induction of a potent immune memory response. Effective targeting of distant metastases after intratumoral administration is also an important finding with significant clinical implications. This novel combination immunotherapy with oncolytic viruses and checkpoint inhibitors following local tumor irradiation is now being translated to a Phase I proof-of-concept clinical trial in non-Hodgkin's lymphoma at our institution.

